# Telomere length and metabolic syndrome traits: A Mendelian randomisation study

**DOI:** 10.1111/acel.13445

**Published:** 2021-07-27

**Authors:** Nellie Y. Loh, Raymond Noordam, Constantinos Christodoulides

**Affiliations:** ^1^ Oxford Centre for Diabetes, Endocrinology and Metabolism Radcliffe Department of Medicine University of Oxford Oxford UK; ^2^ Department of Internal Medicine Section of Gerontology and Geriatrics Leiden University Medical Center Leiden The Netherlands

**Keywords:** adipose tissue, ageing, GWAS, Mendelian randomisation, metabolic syndrome, telomeres

## Abstract

Observational studies have revealed associations between short leucocyte telomere length (LTL), a TL marker in somatic tissues and multiple Metabolic Syndrome (MetS) traits. Animal studies have supported these findings by showing that increased telomere attrition leads to adipose tissue dysfunction and insulin resistance. We investigated the associations between genetically instrumented LTL and MetS traits using Mendelian Randomisation (MR). Fifty‐two independent variants identified at FDR<0.05 from a genome‐wide association study (GWAS) including 78,592 Europeans and collectively accounting for 2.93% of LTL variance were selected as genetic instruments for LTL. Summary‐level data for MetS traits and for the MetS as a binary phenotype were obtained from the largest publicly available GWAS and two‐sample MR analyses were used to estimate the associations of LTL with these traits. The combined effect of the genetic instruments was modelled using inverse variance weighted regression and sensitivity analyses with MR‐Egger, weighted‐median and MR‐PRESSO were performed to test for and correct horizonal pleiotropy. Genetically instrumented longer LTL was associated with higher waist‐to‐hip ratio adjusted for body mass index (*β* = 0.045 SD, SE = 0.018, *p* = 0.01), raised systolic (*β* = 1.529 mmHg, SE = 0.332, p = 4x10^−6^) and diastolic (*β* = 0.633 mmHg, SE = 0.222, *p* = 0.004) blood pressure, and increased MetS risk (OR = 1.133, 95% CI 1.057–1.215). Consistent results were obtained in sensitivity analyses, which provided no evidence of unbalanced horizontal pleiotropy. Telomere shortening might not be a major driver of cellular senescence and dysfunction in human adipose tissue. Future experimental studies should examine the mechanistic bases for the links between longer LTL and increased upper‐body fat distribution and raised blood pressure.

## Graphical abstract

Observational studies have highlighted associations between short leucocyte telomere length, a telomere length marker in somatic tissues and multiple metabolic syndrome traits. We examined the associations between genetically instrumented leucocyte telomere length and metabolic syndrome traits using Mendelian randomisation. Genetically instrumented longer leucocyte telomere length was associated with higher waist‐to‐hip ratio adjusted for body mass index, raised blood pressure and increased metabolic syndrome risk. Telomere shortening might not be a major driver cellular senescence and dysfunction in human adipose tissue.

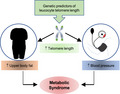

## 1 INTRODUCTION

Metabolic syndrome (MetS) is a cluster of interrelated factors, including abdominal obesity, dyslipidaemia characterised by raised triglycerides and low HDL cholesterol, hyperglycaemia, and hypertension, that increase the risk of type 2 diabetes and cardiovascular atherosclerotic diseases (Alberti et al., 2009). Chronological age is one of the strongest predictors for the development of the MetS and is thought to reflect the impact of biological ageing (Hamczyk et al., 2020). Regardless of the defining criteria used, the prevalence of the MetS is high and rising in both western and developing countries. For example, the prevalence of the MetS in the adult US population between 2011–2016 was estimated to be around 35% and significantly increased with advancing age, being 19.5% among subjects aged 20 to 39 years and 48.6% among those aged 60 years or older (Hirode & Wong, 2020). Insulin resistance is the central hallmark and thought to be the main driver of the MetS and accumulating evidence suggests that it results from inadequate subcutaneous adipose (AT) storage capacity in the face of continuous, excess energy intake resulting in ectopic fat deposition in visceral AT, muscle, liver, pancreas, and the vasculature (Mann & Savage, 2019).

Telomeres are DNA‐protein complexes that cap the ends of eukaryotic chromosomes and function to promote chromosomal stability. Telomere length (TL) varies considerably between subjects and is genetically determined, with heritability estimates between 44%–86% (Li et al., 2020). Telomeres shorten progressively with each cell division and thus TL reflects the amount of cellular turnover within an individual. Accelerated telomere attrition might also occur due to increased exposure to oxidative stress and chronic low‐grade inflammation, both of which are considered important drivers of biological ageing (Beckman & Ames, 1998; Finch & Crimmins, 2004; Mather et al., 2011). Shortened telomeres ultimately reach a critical length, which leads to loss of genome integrity (i.e. DNA damage) with ensuing loss of replicative capacity and cell senescence or apoptosis. This in turn results in compromised tissue stem and progenitor cell function, tissue atrophy and functional decline (Jaskelioff et al., 2011; Sahin & Depinho, 2010). Indeed, short‐term reactivation of endogenous telomerase, the enzyme responsible for telomere maintenance, in adult mice with short dysfunctional telomeres led to reversal of neurodegeneration and tissue atrophy in several other organs including the testes, spleen and intestines (Jaskelioff et al., 2011). As such, telomeres have been proposed as markers of the biological ageing process.

TL is generally measured in leucocytes due to the easy accessibility of these cells in peripheral blood. Nonetheless, as TL within individuals is generally strongly correlated across tissues (Daniali et al., 2013; Demanelis et al., 2020; Gurung et al., 2020), it is thought that leucocyte measures also serve as a marker of TL in other tissues. Cross‐sectional epidemiological studies have demonstrated positive associations between short leucocyte TL (LTL) and multiple traits of the MetS (Monickaraj et al., 2012; Rehkopf et al., 2016; Revesz et al., 2014; Verhulst et al., 2016), as well as risk of coronary artery disease (CAD) (Haycock et al., 2014). In addition, longitudinal data (Cheng et al., 2020; Revesz et al., 2014; Verhulst et al., 2016), have highlighted associations between short baseline LTL and worse metabolic and cardiovascular disease (CVD) outcomes at follow up, although these findings have not been replicated in all studies (Brouilette et al., 2007; Revesz et al., 2018). Nonetheless, results from observational studies may be subject to residual confounding and/or reverse causation bias and cannot infer causality with any degree of certainty. Indeed, with some exceptions (Verhulst et al., 2016), directionally opposite associations between baseline prevalence of CVD risk factors and increased telomere attrition have also been reported (Gardner et al., 2005; Huzen et al., 2014; Revesz et al., 2015, 2018). Consequently, whether short TL is a cause or consequence of the MetS remains unknown. Related to this, whether stem cell senescence due to TL shortening in subcutaneous AT contributes to the upper‐body fat redistribution and metabolic dysfunction associated with ageing (Hirode & Wong, 2020) is unclear. These are important questions in determining whether telomere therapeutics (Nagpal et al., 2020) have a place in the treatment of cardiometabolic disorders, particularly in the elderly. Finally, whether the link between short LTL and increased CAD risk reported in observational (Haycock et al., 2014) and genetic epidemiology (Codd et al., 2013; Kuo et al., 2019; Li et al., 2020) studies is driven by or is independent of conventional CVD risk factors is uncertain. In this respect, whilst a meta‐analysis of observational data concluded that the inverse association between LTL and risk of CAD was independent of established vascular risk factors (Haycock et al., 2014), the largest GWAS on LTL conducted to date, produced suggestive evidence of a shared genetic architecture between short LTL and dyslipidemia (Li et al., 2020).

Mendelian randomisation (MR) is an epidemiological technique using genetic variants as instrumental variables for exposures such as TL. Because genotypes are randomly allocated at conception and are therefore not generally susceptible to reverse causation bias and confounding, in contrast to conventional epidemiological methods, MR can facilitate robust causal inference (Davies et al., 2018). Here we conducted a MR study to determine the association between genetically instrumented LTL and development of the MetS. We hypothesised that shorter telomeres would be causally associated with a higher prevalence of metabolic dysfunction.

## 2 RESULTS

We used 52 independent variants identified at FDR <0.05 as genetic instruments for LTL (Table S1). These were discovered in a recent GWAS meta‐analysis of LTL from 78,592 subjects of European descent and collectively explained 2.93% of the total proportion of LTL variance (Li et al., 2020). Summary‐level data for the different components of the MetS were obtained from the largest publicly available GWAS meta‐analyses for anthropometric (*n* = up to 694,649 subjects) (Pulit et al., 2019), glycaemic (*n* = up to 151,188) (Lagou et al., 2021), lipid (*n* = up to 188,577 subjects) (Willer et al., 2013), and blood pressure (BP) (*n* = up to 757,601 subjects) (Evangelou et al., 2018) traits conducted in Europeans. In the case of lipid traits, we only had access to metadata from a multi‐ancestry GWAS, which nonetheless comprised over 95% European subjects. We also accessed summary statistics from a GWAS of the MetS as a binary trait conducted in the UK Biobank (UKBB) (*n* = 291,107 subjects) (Lind, 2019). Two‐sample MR analyses were subsequently used to estimate the associations of LTL with the MetS and its components.

Using inverse variance weighted (IVW) MR, genetically instrumented longer LTL was associated with higher waist‐to‐hip ratio adjusted for body mass index (WHRadjBMI) (*β* = 0.045 SD, SE = 0.018, *p* = 0.01), higher waist circumference adjusted for BMI (*β* = 0.055 SD, SE = 0.024, *p* = 0.02), increased systolic BP (*β* = 1.529 mm Hg, SE = 0.332, p = 4 × 10^−6^), raised diastolic BP (*β* = 0.633 mmHg, SE = 0.222, *p* = 0.004), and higher odds ratio of developing the MetS (OR = 1.133, 95% CI 1.057–1.215) (Table 1). No significant associations between longer LTL and any other MetS traits were detected.

**TABLE 1:** Two sample MR (IVW) estimates of effects of telomere length on anthropometric, cardiovascular, and metabolic measures. LTL was instrumented using 52 independent variants identified at FDR <0.05 (Li et al., 2020)

Outcomes	*n* instruments	Beta (SE)	p‐value	MR‐Egger test^a^ p‐val	MR‐PRESSO Global test^a^ *p*‐val	Outcome dataset
Anthropometry						
BMI (SD)	46	−0.008 (0.015)	0.6	0.2	**<10^−4^**	Pulit et al., 2019 (PMID**:** 30239722)
WHRadjBMI (SD)	46	0.045 (0.018)	**0.01**	0.9	**<10^−4^**	Pulit et al., 2019 (PMID**:** 30239722)
Waist circumference (adjBMI) (SD)	30	0.055 (0.024)	**0.02**	0.7	0.4	ieu‐a−67 (PMID:25673412)
Glucose homeostasis						
Fasting blood glucose (units)	33	0.025 (0.015)	0.1	0.4	0.9	Lagou et al., 2021 (PMID: 33402679)
Fasting blood insulin (units)	32	−0.015 (0.025)	0.5	**0.03**	**0.007**	Lagou et al., 2021 (PMID: 33402679)
Lipid						
Triglycerides (SD)^b^	26	0.001 (0.030)	1	0.8	0.7	ieu‐a−302 (PMID: 24097068)
HDL cholesterol (SD)^b^	26	0.002 (0.043)	1	0.7	**0.002**	ieu‐a−299 (PMID: 24097068)
Cardiovascular						
Systolic BP (mmHg)	45	1.529 (0.332)	**4 × 10^−6^**	0.1	**<10^−4^**	ieu‐b−38 (PMID: 30224653)
Diastolic BP (mmHg)	45	0.633 (0.222)	**0.004**	**0.04**	**<10^−4^**	ieu‐b−39 (PMID: 30224653)
Metabolic syndrome^c^	47	1.133 (1.057, 1.215)	**0.0005**	0.2	**0.006**	Lind (2019) (PMID: 31589552)

Note

Results are presented as beta estimates with SE. Results were retrieved using the IVW method with the assumption of no bias by directional horizontal pleiotropy.

Bold values indicate *p* < 0.05.

^a^ The MR‐Egger and MR‐PRESSO Global tests were used to detect the presence of pleiotropy.

^b^ Outcome GWAS summary statistics were derived from a multi‐ancestry study (>95% European participants). GWAS summary statistics from subjects of European only ancestry are not available.

^c^ MR results for the metabolic syndrome presented as odds ratio with 95% confidence interval.

**FIGURE 1:**
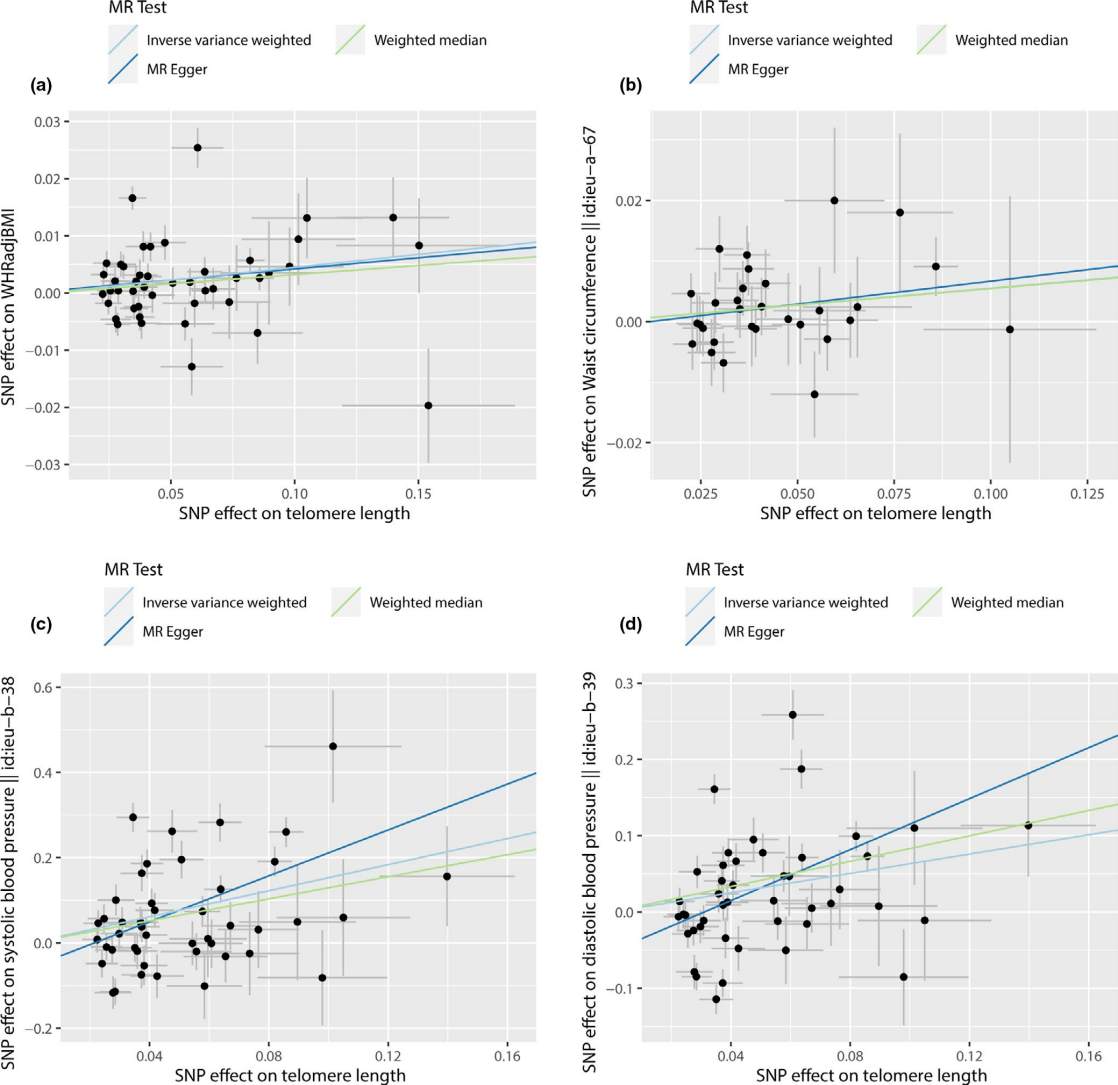
Scatter plots showing the relationship of genetically instrumented LTL on the *x*‐axis, against the following outcomes on the *y*‐axis: (a) WHRadjBMI (n = 46 SNP instruments), (b) waist circumference adjusted for BMI (*n* = 30 SNP instruments), (c) systolic blood pressure (*n* = 45 SNP instruments) and (d) diastolic blood pressure (*n* = 45 SNP instruments). Data points and error bars are betas ± SEs from the respective GWAS summary statistics

To detect violations of MR assumptions, we also conducted sensitivity analyses using MR‐Egger, weighted‐median MR, and MR‐PRESSO (Table S2). All three methods demonstrated consistent directions and similar effect estimates to IVW (Figure 1, Table S2). Furthermore, MR‐Egger regression did not detect evidence of directional pleiotropy for any of the outcomes except for diastolic BP and MetS (binary) which, nonetheless had no major influence on the effect estimate (p for pleiotropy from MR‐Egger = 0.04). On the other hand, MR‐PRESSO revealed evidence of pleiotropic variants for all significant associations, BMI‐adjusted waist circumference aside (p for presence of outliers from MR‐PRESSO<10^−4^) (Table 1). However, removal of these outlier variants made no changes to the significance or interpretation of the results derived using IVW regression (Table S2). Finally, restricting the SNPs included in the LTL genetic instrument to the 21 lead variants with genome‐wide significance produced consistent MR estimates for all outcomes (Table S3).

## 3 DISCUSSION

We have investigated the relationship between LTL and the MetS and its component traits using MR. Contrary to our hypothesis, our study revealed that genetically predicted longer LTL was associating with a higher risk of developing the MetS, which appears to be primarily driven by causal links between longer LTL and raised BP, as well as upper‐body fat distribution. We did not identify any evidence favouring causal associations between LTL and any other constituent parts of the MetS. These findings highlight that (1) genetically instrumented LTL is not associated with insulin resistance, which can account for all the features (Mann & Savage, 2019) and is thought to underpin the pathogenesis of the MetS and (2) there are multiple forms of MetS not all of which are associated with impaired insulin action. Indeed, studies that investigated this relationship showed that only about three quarters of subjects with a diagnosis of MetS were insulin resistant (Cheal et al., 2004). Several studies summarised in a recent review (Smith et al., 2021), reported that senescent cells contribute to the development of AT dysfunction and systemic insulin resistance, at least partly, through the secretion of pro‐inflammatory cytokines and chemokines, known collectively as the senescence‐associated secretory phenotype (SASP). DNA damage, which can result from critical telomere shortening was also demonstrated to directly trigger adipocyte insulin resistance (Vergoni et al., 2016). Finally, short LTL was shown to be associated with increased risk of CAD in both epidemiological and MR studies (Codd et al., 2013; Haycock et al., 2014; Kuo et al., 2019; Li et al., 2020). Viewed in the context of these findings, our results suggest that telomere attrition might not be a major driver of senescence in AT in humans. In this respect, ROS can induce cellular senescence independent of telomere shortening via direct DNA damage and/or chromatin disruption (Campisi, 2005; Shay & Wright, 2005). Furthermore, both ROS and chronic inflammation were demonstrated to cause telomere dysfunction in the absence of shortening (Jurk et al., 2014). Alternatively, or additionally, only a few short telomeres might be sufficient to trigger replicative senescence (Shay & Wright, 2005). Finally, our findings indicate that, the previously observed link between short LTL and CAD risk appears to be independent of conventional cardiovascular risk factors (Smith et al., 2021).

### 3.1 Genetically instrumented LTL and body fat distribution

The finding that genetically instrumented longer LTL was positively associated with upper‐body fat distribution was unexpected because upper‐body obesity, typically characterised by expansion of the visceral fat depot, is a hallmark of dysfunctional subcutaneous AT and is associated with insulin resistance. According to the lipid overflow hypothesis (Virtue & Vidal‐Puig, 2010), when the capacity of adipose progenitors (APs) to generate new adipocytes is exhausted, for example consequent to senescence, fatty acids are inappropriately stored in extra‐adipose tissues including the visceral depot (see Introduction). Accordingly, patients with premature ageing due to rare genetic progeroid syndromes, often develop partial lipodystrophy, characterised by loss of subcutaneous and expansion of visceral AT concomitant with severe insulin resistance (Akinci et al., 2000). Consistent with these findings in humans, mice with short telomeres due to global deficiency of telomerase reverse transcriptase (TERT), the enzyme responsible for telomere maintenance, developed insulin resistance and glucose intolerance on an obesogenic diet (Minamino et al., 2009). This phenotype was coupled with senescent changes and inflammation in AT and was recapitulated by transplantation of AT from TERT deficient mice to wild‐type animals. Knockout of TERT in fat progenitors, also led to proliferative senescence and AP exhaustion, adipocyte hypertrophy and systemic insulin resistance in male but not female mice, which was aggravated by high fat‐diet (Gao et al., 2020). Finally, endothelial cell‐specific progeroid mice developed AT dysfunction and systemic insulin resistance even on a normal chow diet due to the secretion of SASP factors consequent to oxidative stress (Barinda et al., 2020). Interestingly, other metabolic tissues namely muscle, liver, and brown adipose tissue were unaffected. Of note, in parabiosis experiments, endothelial cell‐specific progeroid mice transmitted the metabolic disorders to wild‐type animals (Barinda et al., 2020). Based on the findings described above, we speculate that the preferential upper‐body fat distribution associated with genetically predicted long LTLs is due to healthy AT expansion consequent to enhanced proliferation and adipogenesis of subcutaneous abdominal (rather than visceral) progenitors. In this respect, APs from this depot were shown to have shorter TL than visceral fat progenitors (Lakowa et al., 2015) and thus longer TL might endow these cells with enhanced proliferative capacity. Finally, and provided that LTL is a good surrogate for TL in other tissues as has been reported (see below), our data suggest that TL shortening in AT does not play a major role in the fat redistribution and impaired insulin sensitivity that occurs with ageing.

### 3.2 Genetically instrumented LTL and blood pressure traits

In contrast to fat distribution, the epidemiological data linking LTL and BP traits have been inconsistent, with positive, negative, and mostly null associations reported (Brouilette et al., 2007; Cheng et al., 2020; Huang et al., 2020; Nordfjall et al., 2008; Rehkopf et al., 2016; Revesz et al.,^2014^, ^2015^). This may be because the relationship between LTL and both systolic and diastolic BP is not linear as previously reported (Huang et al., 2020; Rehkopf et al., 2016). On the other hand, mice lacking telomerase activity displayed hypertension due to an increase in plasma endothelin 1 levels, which was, at least in part, driven by increased ROS production (Perez‐Rivero et al., 2006). It is unclear why genetically predicted longer LTL would be causally associated with higher BP as reported herein. One clue may come from an epidemiological study in the Framingham Offspring Cohort, where longer LTL was associated with increased aldosterone‐to‐renin ratio, indicative of aldosterone hypersecretion (Vasan et al., 2009). In this respect, primary hyperaldosteronism is an established cause of hypertension.

### 3.3 Study limitations

Our findings should be interpreted in the context of some limitations. The main limitation is that we studied mean LTL length, which does not necessarily translate to TL in tissues and cells relevant to the development of the MetS, for example adipose stem cells. However, TL within individuals is generally strongly correlated across tissues (Daniali et al., 2013; Demanelis et al., 2020; Gurung et al., 2020). Secondly, our findings are based on data from GWAS conducted in subjects of European ancestry. Hence, our results and conclusions might not extend to other ethnic populations although, evidence from a recent, large, ancestrally diverse GWAS meta‐analysis of glycaemic traits suggests that similar results might also be expected (Chen et al., 2021). Thirdly, two‐sample MR studies assume that the SNP‐exposure (in this case LTL) associations are also present in the outcome dataset(s). Given that both the instrumental variables for LTL, as well as the summary statistics for all outcomes were derived from large GWAS meta‐analyses, we consider this to be a safe assumption. However, we acknowledge that there may be some noise in the causal effect estimates of LTL on the traits investigated herein, depending on the population characteristics (age, BMI, sex, socio‐economic status) of the LTL and the prediction GWAS sets (Mostafavi et al., 2020). Fourthly, our genetic instrument explained only 2.93% of the variance in LTL. However, because all the SNPs associated with LTL in the exposure instrument had an F‐statistic >10 (Table S1), the risk of weak instrument bias is small. On the other hand, the low percentage of LTL variance explained may have led to reduced statistical power in some of the MR analyses. Nonetheless, the sample sizes of all outcome datasets were large (>100,000 participants) and the MR beta estimates small. Thus, any missed associations, except potentially for fasting glucose, are unlikely to be of any biological or clinical significance. Indeed, we had 80% power to detect effect sizes ranging between 0.02–0.05 SDs for BMI, lipid, and glycaemic traits at a significance level of 0.05 (Table S4). In addition, the 52 SNPs used as LTL exposure instruments were derived from a less stringent false discovery rate (FDR) threshold of <0.05. Within this FDR list, 5% of variants (i.e. 2–3 SNPs) are estimated to be false positives. However, near identical results were obtain with the 21 SNP instrument of GWAS significant variants. Furthermore, we were not able to examine the causal role of MetS traits in telomere attrition as we did not have access to summary statistics from any of the LTL GWAS. Finally, we were unable to provide a molecular mechanism to explain the reported observations.

In summary, we provide evidence that genetically instrumented longer LTL is associated with upper‐body fat distribution, raised BP and increased risk of developing the MetS. Future experimental studies should examine the mechanistic basis for these links. In view of previous observational data demonstrating positive associations between short LTL and multiple components of the MetS (Monickaraj et al., 2012; Rehkopf et al., 2016; Revesz et al., 2014; Verhulst et al., 2016), the effects of MetS traits and particularly insulin resistance on LTL should also be investigated using MR. Finally, our data suggest that telomere attrition might not be a major cause of senescence and dysfunction in AT in humans.

## 4 EXPERIMENTAL PROCEDURES

We conducted a two‐sample MR study to investigate the relationships between LTL and measures of adiposity (BMI, WHRadjBMI, and waist circumference adjusted for BMI), fasting glucose homeostasis (fasting glucose, fasting insulin), fasting lipids (triglycerides and HDL cholesterol), and blood pressure (systolic BP, diastolic BP). Genetic instruments for TL were selected from a genome‐wide meta‐analysis for LTL (Table S1, (Li et al., 2020), *n* = 52 instruments, FDR<0.05; GWAS significant (p < 5 × 10^−8^), n = 21 instruments, based on *N* of up to 78,592 individuals of European descent). For outcome data, we used summary‐level results from meta‐analyses of GWAS for obesity and body fat distribution in the UKBB and GIANT (BMI and WHRadjBMI, based on *N* of up to 694,649 individuals of European ancestry (https://github.com/lindgrengroup/fatdistnGWAS) (Pulit et al., 2019), and GWAS summary statistics from the GIANT consortium (waist circumference adjusted for BMI, based on *N* of up to 231,353 individuals of European descent) (MRBase [app.mrbase.org], ieu‐a‐67) (Shungin et al., 2015); the European‐based analyses of the Meta‐Analyses of Glucose and Insulin‐related traits Consortium (MAGIC) (fasting glucose and fasting insulin, based on *N* of up to 151,188 individuals of European ancestry without diabetes mellitus) (https://magicinvestigators.org/ downloads/) (Lagou et al., 2021); the Global Lipids Genetics Consortium (GLGC; fasting lipid traits, based on *N* of up to 188,577 subjects of European [95%], East Asian, South Asian and African ancestry) (MRBase, ieu‐a‐299, ieu‐a‐302) (Willer et al., 2013); and the International Consortium for Blood Pressure (BP phenotypes, based on data from *N* = 757,601 individuals of European descent) (MRBase, ieu‐b‐38, ieu‐b‐39) (Evangelou et al., 2018). For the MetS as defined by the harmonised NCEP criteria (Alberti et al., 2009), we used publicly available GWAS summary statistics from the UKBB (https://www.ukbiobank.ac.uk) (based on *N* = 291,107 individuals of British descent and European ancestry). Individuals who met three of the following five criteria were defined as having MetS: BP ≥130/85 mmHg or antihypertensive treatment, serum glucose ≥6.1 mmol/L or antidiabetic treatment, serum triglycerides ≥1.7 mmol/L, waist circumference >102 cm in men and >88 cm in women, HDL cholesterol <1.0 mmol/L in men and <1.3 mmol/L in women (Lind, 2019).

We used the IVW approach for two‐sample MR analyses (Burgess et al., 2013). The IVW MR estimate assumes that all instruments included in the analyses are valid, affect the outcome only through the exposure, and do not associate with any confounders. However, since this is often not the case, we performed sensitivity analyses including the MR‐Egger regression, weighted‐median estimator and MR‐PRESSO (MR Pleiotropy RESidual Sum and Outlier). MR‐Egger does not force the regression line through the intercept and is therefore able to test for the presence of directional pleiotropy (Bowden et al., 2015). The weighted‐median estimator assumes that at least 50% of the genetic instruments are valid (Bowden et al., 2016). MR‐PRESSO (https://github.com/rondolab/MR‐PRESSO) detects the presence of variant effect sizes that are outliers and corrects pleiotropy via outlier removal (Verbanck et al., 2018). Analyses were conducted using the TwoSampleMR package (v0.5.5) and MRPRESSO package (v1.0) implemented in R (v3.6.3) statistical software (Hemani et al., 2018; Verbanck et al., 2018; Yavorska & Burgess, 2017) according to the TwoSampleMR Guide (https://mrcieu.github.io/TwoSampleMR/), and MRPRESSO vignette, respectively.

## CONFLICTS OF INTEREST

The authors have no conflicts of interest to declare.

## AUTHOR CONTRIBUTIONS

Conceptualisation: C.C.; Investigation: all authors; Writing, review, and editing: all authors.

## Supporting information

Supplementary Material

## Data Availability

Full GWAS summary statistics for the exposure and outcome data used herein can be found at https://www.ebi.ac.uk/gwas (waist circumference adjusted for BMI, lipid traits, BP traits and the MetS; study accessions GCST009001‐GCST010000 & GCST009602), https://github.com/lindgrengroup/fatdistnGWAS (BMI and WHRadjBMI) and https://magicinvestigators.org/ downloads/ (fasting glucose and fasting insulin).
