# Supplementary material for: Telomere length and metabolic syndrome traits: A Mendelian randomisation study
Source: Aging Cell. 2021 Jul 27;20(8):e13445. doi: 10.1111/acel.13445 (PMC8373272; doi:10.1111/acel.13445)
Supplement: Supplementary file 1 — Supplementary Material [file ACEL-20-e13445-s001.docx]

**Supplementary Information**

Telomere length and Metabolic Syndrome traits: a Mendelian Randomisation study.

Nellie Y. Loh , Raymond Noordam , and Constantinos Christodoulides .

Table S1. SNPs used for the LTL instruments in the Mendelian Randomisation analyses.

| SNP | effect_allele | other_allele | EAF | Beta | SE | p | n | F-statistic |
| --- | --- | --- | --- | --- | --- | --- | --- | --- |
| rs10936600 | T | A | 0.243 | -0.086 | 0.006 | 6x10^-51^ | 80402.0 | 225.3 |
| rs7705526 | A | C | 0.328 | 0.082 | 0.006 | 5x10^-45^ | 64656.3 | 198.3 |
| rs2853677 | A | G | 0.592 | -0.064 | 0.005 | 3x10^-31^ | 66348.1 | 135.1 |
| rs4691895 | C | G | 0.783 | 0.058 | 0.006 | 1x10^-21^ | 77751.1 | 91.0 |
| rs9419958 | C | T | 0.862 | -0.064 | 0.007 | 5x10^-19^ | 79673.7 | 79.5 |
| rs75691080 | T | C | 0.091 | -0.067 | 0.009 | 6x10^-14^ | 73299.7 | 56.5 |
| rs59294613 | A | C | 0.293 | -0.041 | 0.005 | 1x10^-13^ | 77807.4 | 55.1 |
| rs8105767 | G | A | 0.289 | 0.039 | 0.005 | 5x10^-13^ | 80103.0 | 52.1 |
| rs73624724 | C | T | 0.129 | 0.051 | 0.007 | 6x10^-12^ | 79451.3 | 47.3 |
| rs3219104 | C | A | 0.830 | 0.042 | 0.006 | 9x10^-11^ | 82701.8 | 42.0 |
| rs932827 | T | C | 0.238 | -0.037 | 0.006 | 3x10^-10^ | 75271.4 | 39.5 |
| rs2736176 | C | G | 0.313 | 0.034 | 0.005 | 3x10^-10^ | 74733.4 | 39.4 |
| rs3785074 | G | A | 0.263 | 0.035 | 0.006 | 4x10^-10^ | 78946.7 | 38.9 |
| rs7194734 | T | C | 0.782 | -0.037 | 0.006 | 7x10^-10^ | 79221.3 | 38.1 |
| rs34978822 | G | C | 0.015 | -0.140 | 0.023 | 7x10^-10^ | 64578.6 | 38.0 |
| rs34991172 | G | T | 0.068 | -0.061 | 0.010 | 6x10^-9^ | 69563.3 | 33.8 |
| rs228595 | A | G | 0.417 | -0.028 | 0.005 | 1x10^-8^ | 79131.2 | 32.2 |
| rs2302588 | C | G | 0.100 | 0.048 | 0.008 | 2x10^-8^ | 75515.0 | 31.9 |
| rs13137667 | C | T | 0.959 | 0.077 | 0.014 | 2x10^-8^ | 65743.6 | 31.2 |
| rs55749605 | A | C | 0.579 | -0.037 | 0.007 | 2x10^-8^ | 44477.5 | 31.2 |
| rs62053580 | G | A | 0.169 | -0.039 | 0.007 | 4x10^-8^ | 68784.9 | 30.2 |
| rs754017156 | NA | GGGCCCGGGCCCGGGCCC/GGGCCC/GGGCCCGGGCCC/GGGCCCGGGCCCGGGCCCGGGCCC/GGGCCCGGGCCCGGGCCCGGGCCCGGGCCCGGGCCC | 0.165 | 0.047 | 0.009 | 8x10^-8^ | 45835.0 | 28.9 |
| rs12909131 | T | C | 0.231 | -0.031 | 0.006 | 1x10^-7^ | 80706.5 | 28.1 |
| rs1744757 | T | C | 0.851 | 0.036 | 0.007 | 1x10^-7^ | 82222.6 | 27.8 |
| rs2124616 | A | G | 0.140 | -0.037 | 0.007 | 2x10^-7^ | 78571.2 | 27.3 |
| rs2613954 | T | C | 0.886 | -0.038 | 0.008 | 1x10^-6^ | 78132.7 | 23.7 |
| rs12065882 | G | A | 0.208 | 0.030 | 0.006 | 1x10^-6^ | 77170.9 | 23.3 |
| rs2386642 | A | G | 0.673 | -0.026 | 0.005 | 1x10^-6^ | 78324.5 | 23.2 |
| rs56810761 | T | C | 0.270 | 0.027 | 0.006 | 1x10^-6^ | 75729.8 | 23.2 |
| rs62365174 | G | A | 0.088 | -0.054 | 0.011 | 1x10^-6^ | 47138.2 | 23.1 |
| rs112655343 | T | C | 0.102 | 0.043 | 0.009 | 2x10^-6^ | 65703.2 | 22.4 |
| rs55710439 | T | C | 0.014 | 0.105 | 0.022 | 3x10^-6^ | 69379.6 | 22.1 |
| rs11640926 | G | T | 0.139 | 0.056 | 0.012 | 3x10^-6^ | 28512.8 | 21.9 |
| rs60160057 | A | G | 0.211 | -0.029 | 0.006 | 3x10^-6^ | 76458.6 | 21.7 |
| rs117536281 | G | A | 0.034 | 0.085 | 0.018 | 3x10^-6^ | 43901.3 | 21.6 |
| rs7510583 | G | A | 0.290 | 0.035 | 0.007 | 3x10^-6^ | 42136.9 | 21.6 |
| rs59192843 | G | T | 0.059 | 0.065 | 0.014 | 4x10^-6^ | 43632.0 | 21.5 |
| rs57415150 | A | G | 0.042 | -0.058 | 0.013 | 4x10^-6^ | 76209.6 | 21.4 |
| rs6038821 | T | A | 0.038 | 0.060 | 0.013 | 4x10^-6^ | 78795.1 | 21.3 |
| rs144204502 | T | C | 0.014 | -0.090 | 0.020 | 5x10^-6^ | 90239.0 | 20.9 |
| rs6107615 | C | T | 0.422 | -0.023 | 0.005 | 6x10^-6^ | 79235.8 | 20.7 |
| rs9972513 | T | C | 0.281 | 0.025 | 0.005 | 6x10^-6^ | 80585.1 | 20.6 |
| rs117037102 | T | C | 0.018 | 0.098 | 0.022 | 7x10^-6^ | 58251.0 | 20.2 |
| rs7276273 | C | A | 0.007 | -0.150 | 0.033 | 7x10^-6^ | 58815.8 | 20.2 |
| rs11665818 | A | G | 0.195 | 0.028 | 0.006 | 7x10^-6^ | 80994.7 | 20.2 |
| rs3213718 | T | C | 0.583 | 0.022 | 0.005 | 7x10^-6^ | 79728.4 | 20.1 |
| rs112347796 | AG | AGAG | 0.049 | 0.069 | 0.015 | 7x10^-6^ | 43935.8 | 20.1 |
| rs143276018 | C | T | 0.018 | -0.102 | 0.023 | 9x10^-6^ | 51875.2 | 19.7 |
| rs201375979 | - | TA | 0.317 | 0.033 | 0.007 | 9x10^-6^ | 39878.3 | 19.7 |
| rs7311314 | A | G | 0.317 | 0.024 | 0.005 | 9x10^-6^ | 75916.0 | 19.6 |
| rs35675808 | G | C | 0.028 | 0.074 | 0.017 | 1x10^-5^ | 64171.8 | 19.6 |
| rs117610974 | G | C | 0.009 | -0.154 | 0.035 | 1x10^-5^ | 42498.8 | 19.4 |

n = Estimated effective sample size as calculated by Li et al., 2020.

Table S2. Two sample MR (MR-Egger, Weighted-median, MR-PRESSO) estimates of effects of telomere length on anthropometric, cardiovascular, and metabolic measures. LTL was instrumented using 52 independent variants identified at FDR < 0.05 (Li et al., 2020).

| Outcomes | *n* instruments | MR-Egger | Weighted- median | MR-PRESSO^b^ | Outcome dataset |
| --- | --- | --- | --- | --- | --- |
| **Anthropometry** |  |  |  |  |  |
| BMI (SD) | 46 | -0.047 (0.034) | -0.012 (0.014) | -0.002 (0.013) | Pulit et al. 2019  (PMID**: 30239722)** |
| WHRadjBMI (SD) | 46 | 0.039 (0.041) | 0.032 (0.015)* | 0.028 (0.013)* | Pulit et al. 2019  (PMID**: 30239722)** |
| Waist circumference  (adjBMI) (SD) | 30 | 0.076 (0.066) | 0.054 (0.036) | NA | ieu-a-67  (PMID:25673412) |
| **Glucose Homeostasis** |  |  |  |  |  |
| Fasting blood glucose (units) | 33 | -0.004 (0.038) | 0.009 (0.023) | NA | Lagou et al. 2021 (**PMID: 33402679**) |
| Fasting blood insulin (units) | 32 | -0.134 (0.056)* | -0.045 (0.032) | -0.028 (0.023) | Lagou et al. 2021 (**PMID: 33402679**) |
| **Lipid** |  |  |  |  |  |
| ^a^Triglycerides (SD) | 26 | 0.027 (0.091) | -0.003 (0.042) | NA | ieu-a-302  (PMID: 24097068) |
| ^a^HDL cholesterol (SD) | 26 | 0.050 (0.133) | 0.003 (0.050) | NA | ieu-a-299  **(PMID:**24097068) |
| **Cardiovascular** |  |  |  |  |  |
| Systolic BP (mmHg) | 45 | 2.690 (0.809)** | 1.293 (0.268)^#^ | 1.104 (0.243)^#^ | ieu-b-38  **(PMID:**30224653) |
| Diastolic BP (mmHg) | 45 | 1.670 (0.529)** | 0.831 (0.143)^#^ | 0.643 (0.127)^#^ | ieu-b-39  (PMID: 30224653) |
| **^c^Metabolic Syndrome** | 47 | 1.246 (1.055, 1.472)* | 1.137 (1.044, 1.239)** | 1.147 (1.074, 1.225)** | Lind (2019)  (PMID: **31589552)** |

Results are presented as beta estimates with SE.

^a^Outcome GWAS summary statistics from a multi-ancestry study (>95% European participants). GWAS summary statistics from individuals of European only ancestry are not available.

^b^Outlier-corrected. NA, not available.

^c^MR results for the metabolic syndrome presented as odds ratio with 95% confidence interval.

*p<0.05, **p<0.005, ^#^p<10^-4^.

Table S3. Two sample MR (IVW) estimates of effects of telomere length on anthropometric, cardiovascular, and metabolic measures. LTL was instrumented using 21 independent variants identified at GWAS significance (Li et al., 2020).

| Outcomes | *n* instruments (GWAS significant) | Beta (SE) | p-value | MR-Egger test ^a^p-val | Outcome dataset |
| --- | --- | --- | --- | --- | --- |
| **Anthropometry** |  |  |  |  |  |
| BMI (SD) | 20 | -0.029 (0.019) | 0.1 | 0.5 | Pulit et al. 2019  (PMID: **30239722)** |
| WHRadjBMI (SD) | 20 | 0.064 (0.028) | **0.02** | 1 | Pulit et al. 2019  (PMID: **30239722)** |
| Waist circumference  (adjBMI) (SD) | 14 | 0.073 (0.029) | **0.01** | 1 | ieu-a-67  (PMID:25673412) |
| **Glucose Homeostasis** |  |  |  |  |  |
| Fasting blood glucose (units) | 16 | 0.022 (0.020) | 0.26 | 0.15 | Lagou et al. 2021 (**PMID: 33402679)** |
| Fasting blood insulin (units) | 15 | -0.049 (0.038) | 0.2 | 0.3 | Lagou et al. 2021 (**PMID: 33402679**) |
| **Lipid** |  |  |  |  |  |
| ^b^Triglycerides (SD) | 12 | 0.035 (0.042) | 0.4 | 0.7 | ieu-a-302  (PMID: 24097068) |
| ^b^HDL cholesterol (SD) | 12 | 0.038 (0.066) | 0.6 | 0.6 | ieu-a-299  **(PMID:**24097068) |
| **Cardiovascular** |  |  |  |  |  |
| Systolic BP (mmHg) | 21 | 2.109 (0.464) | **5x10^6^** | 0.4 | ieu-b-38  **(PMID:**30224653) |
| Diastolic BP (mmHg) | 21 | 0.972 (0.344) | **0.005** | 0.2 | ieu-b-39  (PMID: 30224653) |
| **^c^Metabolic syndrome** | 21 | 1.122 (1.026, 1.227) | **0.01** | 0.05 | Lind (2019)  (PMID: **31589552)** |

Results are presented as beta estimates with SE.

Results were retrieved using the IVW method with the assumption of no bias by directional horizontal pleiotropy.

^a^The MR-Egger test was used to detect the presence of potential directional horizontal pleiotropy.

^b^Outcome GWAS summary statistics from a multi-ancestry study (>95% European participants). GWAS summary statistics from individuals of European only ancestry are not available.

^c^MR results for the metabolic syndrome presented as odds ratio with 95% confidence interval.

Table S4. Power calculation for Mendelian Randomisation studies using LTL genetic instruments.

| Outcomes | Sample size | Proportion of women | Proportion of cases | *R*^2^_xz_ | OR (IVW) | Beta (IVW) | Variance (exposure) | Variance  (outcome) | Power | | Betas, at 80% power | |
| --- | --- | --- | --- | --- | --- | --- | --- | --- | --- | --- | --- | --- |
|  |  |  |  |  |  |  |  |  | α = 0.05 | α = 0.01 | α = 0.05 | α = 0.01 |
| MetS | 291,107 | 0.47 | 0.205 | 0.0293 | 1.133 |  |  |  | 100% | 99% | OR=1.076 | OR=1.093 |
| BMI | 694,649 | 0.55 |  | 0.0293 |  | -0.008 | 1 | 1 | 21% | 8% | 0.020 | 0.024 |
| WHRadjBMI | 694,649 | 0.55 |  | 0.0293 |  | 0.045 | 1 | 1 | 100% | 100% | 0.020 | 0.024 |
| WCadjBMI | 231,353 | 0.55 |  | 0.0293 |  | 0.055 | 1 | 1 | 99% | 97% | 0.034 | 0.042 |
| Fasting glucose | 151,188 | 0.52 |  | 0.0293 |  | 0.025 | 1 | 1 | 38% | 18% | 0.042 | 0.052 |
| Fasting insulin | 105,056 | 0.51 |  | 0.0293 |  | -0.015 | 1 | 1 | 13% | 4% | 0.051 | 0.062 |
| Triglycerides | 177,861 | 0.48 |  | 0.0293 |  | 0.001 | 1 | 1 | 5% | 1% | 0.039 | 0.048 |
| HDL cholesterol | 187,167 | 0.48 |  | 0.0293 |  | 0.002 | 1 | 1 | 5% | 1% | 0.038 | 0.046 |
| Systolic BP | 757,601 | 0.55 |  | 0.0293 |  | 1.529 | 1 | 1 | 100% | 100% | 0.019 | 0.023 |
| Diastolic BP | 757,601 | 0.55 |  | 0.0293 |  | 0.633 | 1 | 1 | 100% | 100% | 0.019 | 0.023 |

Power calculations were performed using the online web tool mRnd (<https://shiny.cnsgenomics.com/mRnd/>) (Brion, Shakhbazov, & Visscher, 2013).

OR, odds ratio. *R*^2^_xz_, proportion of variance explained by genetic instruments on exposure (LTL).

**Supplementary Reference**

Brion, M. J., Shakhbazov, K., & Visscher, P. M. (2013). Calculating statistical power in Mendelian randomization studies. *Int J Epidemiol, 42*(5), 1497-1501. doi:10.1093/ije/dyt179
